# Evaluating the Quality of Evidence from a Network Meta-Analysis

**DOI:** 10.1371/journal.pone.0099682

**Published:** 2014-07-03

**Authors:** Georgia Salanti, Cinzia Del Giovane, Anna Chaimani, Deborah M. Caldwell, Julian P. T. Higgins

**Affiliations:** 1 Department of Hygiene and Epidemiology, University of Ioannina School of Medicine, Ioannina, Greece; 2 Statistics Unit, Department of Clinical and Diagnostic Medicine and Public Health, University of Modena and Reggio Emilia, Modena, Italy; 3 School of Social and Community Medicine, University of Bristol, Bristol, United Kingdom; 4 Centre for Reviews and Dissemination, University of York, York, United Kingdom; National Taiwan University, Taiwan

## Abstract

Systematic reviews that collate data about the relative effects of multiple interventions via network meta-analysis are highly informative for decision-making purposes. A network meta-analysis provides two types of findings for a specific outcome: the relative treatment effect for all pairwise comparisons, and a ranking of the treatments. It is important to consider the confidence with which these two types of results can enable clinicians, policy makers and patients to make informed decisions. We propose an approach to determining confidence in the output of a network meta-analysis. Our proposed approach is based on methodology developed by the Grading of Recommendations Assessment, Development and Evaluation (GRADE) Working Group for pairwise meta-analyses. The suggested framework for evaluating a network meta-analysis acknowledges (i) the key role of indirect comparisons (ii) the contributions of each piece of direct evidence to the network meta-analysis estimates of effect size; (iii) the importance of the transitivity assumption to the validity of network meta-analysis; and (iv) the possibility of disagreement between direct evidence and indirect evidence. We apply our proposed strategy to a systematic review comparing topical antibiotics without steroids for chronically discharging ears with underlying eardrum perforations. The proposed framework can be used to determine confidence in the results from a network meta-analysis. Judgements about evidence from a network meta-analysis can be different from those made about evidence from pairwise meta-analyses.

## Introduction

A network meta-analysis produces inferences regarding the relative effectiveness or safety of multiple treatments [1]–[3]. Just as for a traditional meta-analysis of pair-wise comparisons, it is essential to consider the confidence that can be placed in results from a network meta-analysis, and to convey this clearly so that the reader can make an informed judgement about how to use the findings. In this paper we propose an approach to considering the quality of evidence arising from a network meta-analysis, inspired by the methodology developed by the Grading of Recommendations Assessment, Development and Evaluation (GRADE) Working Group. Our proposals are developed based on our experiences of undertaking and interpreting network meta-analyses.

The GRADE approach leads to judgements about the confidence with which an estimate of treatment effect for a particular outcome can be believed, using four levels: high, moderate, low and very low. When the evidence arises from randomized trials – as is usually the case in network meta-analysis – the body of evidence is initially assigned to a high quality rating. Then five components are considered: study limitations, inconsistency, indirectness, imprecision and publication bias. For each component, the quality of the evidence can be maintained or downgraded by up to two levels, subject to a maximum downgrade by three levels (to very low quality) across the five components.

Some authors have applied *ad hoc* modifications of GRADE alongside network meta-analyses. For example, Dumville et al 2012 [4] adapted the five GRADE domains to better address the needs of evaluating evidence across a network of comparisons. In particular, they include a separate category “sensitivity of results” to assess the stability of the network and consider unexplained heterogeneity and inconsistency together, as a single domain on “indirectness/inconsistency”. The GRADE Working Group have addressed the issue of grading for indirect comparisons [5] and have prepared a working paper for network meta-analyses [6].

In our proposal we draw a key distinction between two types of findings from network meta-analysis for a specific outcome: a) effect sizes for pairwise comparisons of treatments (such as odds ratios), and b) a ranking of the treatments. The pairwise effect sizes are estimated using all relevant evidence in the network of treatment comparisons, and may be re-interpreted to aid decision-making, for example using ‘assumed’ and ‘estimated’ risks of an event as in Summary of Findings tables [7]. A list of pairwise effect sizes is particularly informative when one of the treatments is a standard reference treatment such as placebo or no treatment, in which case the list will usefully comprise an effect size for each of the active treatments. The ranking of the treatments should be done using probabilistic methods, for example using ‘rankograms’ or the surface under the cumulative ranking curve (SUCRA), which take into account the estimated effect sizes and their accompanying uncertainty [8].

**Figure 1:**
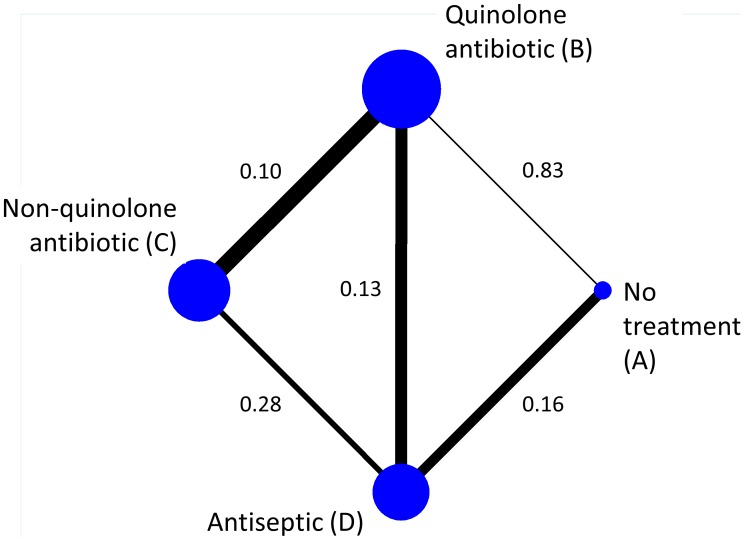
Network of topical antibiotics without steroids for chronically discharging ears. Edges are weighted according to the inverse of the variance of the direct summary ln(OR) (presented along the edges) and nodes are weighted according to the number of studies.

To illustrate the ideas presented in this paper, we use an example network of topical antibiotics without steroids for chronically discharging ears with underlying eardrum perforations [9]. We focus on the outcome of whether or not patients had persistent discharge from the ear after one week, and we measure it using the odds ratio (OR). Figure 1 shows the network of available direct comparisons. In Table 1 we present the number of studies providing direct evidence (OR, 95% and variances) for the five observed comparisons.

**Table 1:** Summary information from direct comparisons of topical antibiotics without steroids for chronically discharging ears.

Comparison	No. studies	Direct evidenceOR (95% CI)	Variance of ln(OR)	I^2^ (p-value)	τ^2^
AB: Quinolone antibiotic vs no treatment	2	0.09 (0.01, 0.51)	0.83	69% (0.07)	1.22
AD: Antiseptic vs no treatment	1	1.42 (0.65, 3.09)	0.16	NE	NE
BC: Non-quinolone antibiotic vs quinolone antibiotic	7	1.46 (0.80, 2.67)	0.10	48% (0.07)	0.31
BD: Antiseptic vs quinolone antibiotic	5	3.47 (1.71, 7.07)	0.13	66% (0.02)	0.39
CD: Antiseptic vs non-quinolone antibiotic	4	1.69 (0.59, 4.83)	0.28	67% (0.03)	0.75

Number of studies, the direct evidence from pairwise meta-analysis (OR, 95% confidence interval and variance) and information about heterogeneity (I^2^ and heterogeneity variance τ^2^).

NE: Not estimable.

## Methodological Considerations and Definitions

### Different types of evidence in a network of treatments

The data underlying a network meta-analysis, as in Figure 1, comprise a series of direct comparisons, providing **direct evidence** on particular pairs of treatments. Treatments that have not been compared directly can be compared indirectly by contrasting effect sizes involving a common comparator [3]; [10]–[12]. For example, in Figure 1, treatments A and C have not been compared directly, but **indirect evidence** is available by contrasting the effect size from the direct AB evidence with the effect size from the direct BC evidence. Alternatively, indirect evidence on AC is available via treatment D (contrasting AD with CD evidence), and also by a longer chain of evidence involving intermediate treatments B and D (e.g. following a route AB–BD–DC through the network). We refer to routes involving a single intermediate treatment as *simple indirect evidence* and routes involving two or more intermediate treatments as *compound indirect evidence*. Indirect comparisons are built on an assumption of **transitivity**, and are fundamental to network meta-analysis. For the transitivity assumption to hold, the studies making different direct comparisons must be sufficiently similar in all respects other than the treatments being compared. When both direct and indirect evidence is available we say that there is **mixed evidence**. A network meta-analysis analyses simultaneously all direct and indirect evidence for all comparisons of the treatments in the network. For each comparison in the network, the inferences from the network meta-analysis may be based on direct evidence alone, indirect evidence alone, or mixed evidence. For example, in Figure 1, there is indirect evidence alone for comparison AC, and mixed evidence for comparisons AB, AD, BC, BD and CD. No comparisons in this network are informed by direct evidence alone.

### Confidence in effect sizes versus confidence in the ranking of treatments

A distinction between the two types of output (pairwise comparisons and overall ranking) is important when assessing our confidence in the evidence that they convey. Ranking measures involve inferences about the network of evidence as a whole, whereas pairwise effect sizes are derived from complex weighted averages of particular sources of direct and indirect evidence, with direct evidence usually contributing more weight. Consider a simple triangular network with high quality evidence for AB but low quality evidence for BC and AC. We might be able to award high confidence to the effect size AB, but only low confidence to the overall treatment ranking.

The aim of this paper is we make suggestions about how to evaluate these two types of output from a network meta-analysis. We consider each component of GRADE separately (study limitations, inconsistency, indirectness, imprecision and publication bias). Then we summarize across all five components to obtain a **confidence in each (pairwise) effect size** and a **confidence in ranking** of treatments. To this end, understanding the flow of information around a network is necessary and we address this in the following two sections.

### The impact of direct evidence on effect sizes and ranking

Judgements about our confidence in an estimated effect size can be made through consideration of the quality of all pieces of evidence that contribute to it. For instance, confidence in the mixed evidence for AB in Figure 1 can be determined by integrating the quality of the direct AB evidence with the quality of the various pieces of indirect evidence linking A and B. In this example, all of the other four direct comparisons contribute directly or indirectly to estimation of the AB comparison when a network meta-analysis is performed. However, the contributions of the five pieces of evidence are not all the same, and are determined by a complicated function of their statistical precision and the network structure. As a general rule, evidence from a direct comparison contributes more than evidence involved in an indirect comparison, and evidence involved in a simple indirect comparison (e.g. comparing A and B via D) contributes more than evidence involved in a compound indirect comparison (e.g. comparing A and B via both C and D). These contributions depend additionally on the amount of information (e.g. number of studies) available. Fortunately, it is possible to determine the contribution of each direct estimate to each estimated effect size from the network meta-analysis, and also the contribution of each direct estimate to the network as a whole [13]. We outline how this works in the next section.

### The contributions matrix: the information contribution of direct evidence to network meta-analysis results

Consider a simple triangular network ABC. We first summarize all AB, AC and BC studies separately to obtain the effect estimates from the direct evidence alone using standard meta-analysis. We denote these as 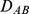, 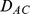 and 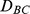 respectively. In a network meta-analysis of the full set of studies, we obtain estimates of the same comparisons based on mixed evidence, each one of which is a different combination of the direct estimates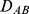, 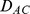 and 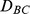. For the simple situation in which each of the direct estimates has the same variance, the network meta-analysis estimate for AB turns out to be 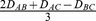. Thus the three direct estimates of AB, AC and BC have absolute weights 2/3, 1/3 and 1/3 or, rescaled as percentages (e.g. dividing them by 4/3), provide contributions of 50%, 25% and 25% respectively. This suggests that judgments about our confidence in network meta-analysis estimates for comparison AB should draw more from the quality of the AB direct evidence and less from the quality of the direct evidence about BC and AC. Deriving the contribution of each direct piece of evidence is more complicated when variances are not equal and when the network structure is complex. Methodology to determine these has recently been proposed [13], and is summarized in Appendix S1. It is based on a frequentist synthesis of information across comparisons; for Bayesian network meta-analyses, the results are not exact but may be regarded as useful approximations.

**Figure 2:**
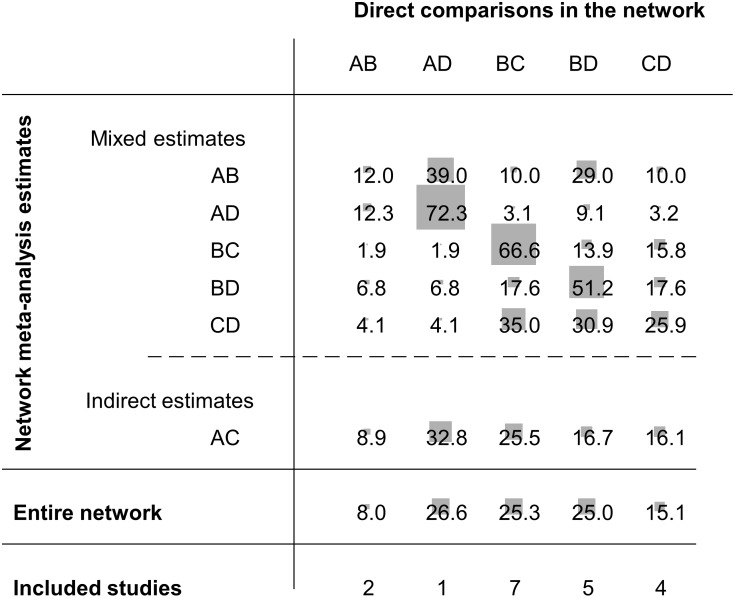
Contributions matrix: percentage contribution of each direct estimate to the network meta-analysis estimates. Rows correspond to network meta-analysis ORs (separated for mixed and indirect evidence) and columns correspond to direct meta-analysis ORs. The contribution of each direct comparison to the total network evidence that provides the ranking of the treatments is presented separately (row named Entire network). The sizes of the boxes are proportional to the percentage contribution of each direct estimate to the network meta-analysis estimates (rows 1–6) and to the entire network (row 7). The last row shows the number of included direct comparisons. The names of the treatments are given in Figure 1.

Consider our example in Figure 1. There are five direct effect estimates, 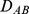, 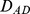, 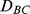, 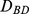 and 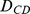. We present these with their associated variances in the third and fourth columns of Table 1. Figure 2 gives the percentage contribution of each direct estimate to each network meta-analysis estimate. Suppose we are interested in the network meta-analysis estimate for AC and we want to understand how each one of the five direct comparisons contributes to this. The **contributions matrix** in Figure 2 shows that we get indirect evidence from AB, AD, BC, BD and CD studies, with contributions 8.9%, 32.8%, 25.5%, 16.7 and 16.1% respectively. We will call these percentage contributions the **contribution of direct evidence to the network meta-analysis effect size** for a particular pairwise comparison. For instance, the contribution of direct evidence BD to the network meta-analysis estimate of AC is 16.7%. These contributions depend on the variances of the direct estimates (see Table 1) and their ‘closeness’ to the target comparison (see Figure 1). For example, the BD direct estimate is more precise than the CD direct estimate (variances 0.13 and 0.28 respectively) so we might expect that the contribution of BD would be greater. However, both BD and CD contribute to the AC network meta-analysis estimate by about the same amount (16.7% and 16.1%). This is because BD is ‘penalized’ for providing compound indirect evidence whereas CD provides simple indirect evidence. The contribution plot can be obtained in STATA (see [14]).

Generally, the largest contribution to each network estimate is provided by the respective direct evidence, but when direct evidence is missing or is imprecise more information is obtained indirectly. These contributions may be interpreted as weights and should be taken into account when evaluating the quality of network evidence for each pairwise comparison.

We can estimate the importance of information from each direct estimate to the entire network as well as to each pairwise comparison. Using the methodology outlined in Appendix S1, we can calculate the weights for each of the five direct comparisons contributing to each NMA estimate. Then, the weights are summed for each direct comparison and then re-expressed as percentages to calculate the percentage contribution of each direct comparison to the full network as shown in the seventh row of Figure 2. We call these contributions the **contribution of direct evidence to the whole network**. These contributions can be used to evaluate the quality of evidence for inferences that relate to the network as a whole, specifically the ranking of treatments. In this example, more importance would be placed on the quality of evidence provided by AD, BC and BD studies and less by the AB or CD studies. The direct comparison AD, although less precise than BD and BC, has the largest contribution to the network because it is the most influential evidence for the AC indirect comparison and provides the largest ‘boosting’ to the imprecisely estimated AB comparison. In summary, direct evidence that contributes a lot of information to a few comparisons or little information to many comparisons turns out to be important. Note that to derive the contributions of each direct estimate we account not only for the precision of each direct comparison but also for the network structure, and the method gives credit to comparisons that are ‘central’ and facilitate indirect comparisons.

## Confidence in Each Effect Size: Evaluating Quality of the Evidence for Each Pairwise Comparison Arising from a Network Meta-Analysis

To determine our confidence in each estimate of effect size from a network meta-analysis, we follow the standard GRADE approach but make some modifications to reflect specific issues in network meta-analysis. These include (i) the key role of indirect comparisons (suggesting a reconsideration of the ‘indirectness’ component of GRADE); (ii) the contributions to each piece of direct evidence to the network meta-analysis estimates of effect size; (iii) the importance of the transitivity assumption to validity of network meta-analysis; and (iv) the possibility of inconsistency between direct evidence and indirect evidence.

### Study limitations

In the GRADE approach, randomized trials are evaluated according to generation of the allocation sequence, concealment of the allocation sequence, blinding, incomplete accounting of participants and outcome events, selective outcome reporting bias and other limitations [15]. The main consideration for study limitations in a network meta-analysis is to ensure that the relative contributions of different sources of direct evidence (which may have different study limitations) are accounted for appropriately. Our proposed procedure is as follows.

- Evaluate each piece of direct evidence in the network and classify it as low, moderate or high risk of bias according to the usual GRADE guidelines.
- For each pairwise network estimate, consider the contribution of all direct estimates feeding into it. For a formal statistical approach to this, we recommend using the contributions matrix.
- Illustrate the risk of bias assessments according to the contributions of each source of direct evidence to each network meta-analysis effect estimate, for example using a bar chart. We conventionally use green, yellow and red to represent low, moderate and high risk of bias.
- For each pairwise comparison, integrate the risk of bias judgements and the respective contributions into a single judgement about study limitations and consider whether to downgrade the quality of the evidence. This can be done informally by interpreting the illustration in step (c). Alternatively, a highly quantitative approach would be to assign numerical scores to each risk of bias judgement (e.g. 0 for low, −1 for moderate and −2 for high risk of bias), and take a weighted average of these using the contribution of each direct estimate to the network estimates from the contributions matrix.

### Application to antibiotics for discharging ears

**Figure 3:**
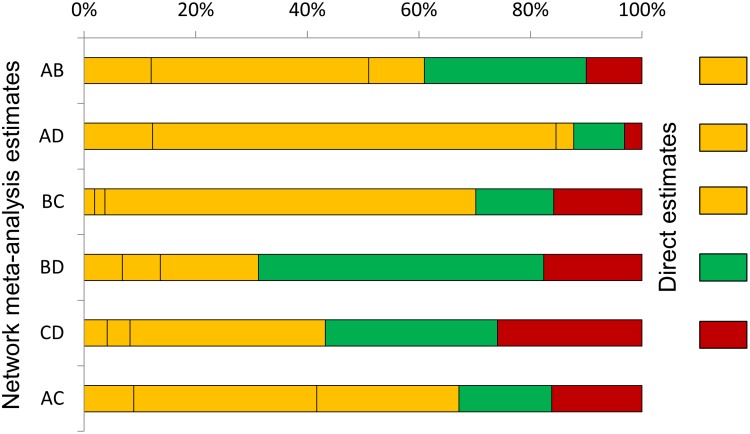
Study limitations for each network estimate for pairwise comparisons of topical antibiotics. Calculations are based on the contributions of direct evidence. The colours represent the risk of bias (green: low, yellow: moderate, red: high). The initial judgements about the risk of bias in the direct estimates are shown on the right side of the figure (there is no direct evidence for AC). The names of the treatments are given in Figure 1.

In our example we have decided that the direct evidence for AD, AB and BC comparisons have moderate risk of bias, direct evidence for the CD comparison has high risk of bias, and only evidence for the BD has low risk of bias. Figure 3 shows the contributions of the direct evidence using the percentage contributions shown in Figure 2, coloured according to our judgements about risk of bias. Examining this plot suggests that it might be appropriate to downgrade only by one level (rather than two levels) the network meta-analysis estimate for the CD comparison, because most information (about 74%) for this comes from studies with moderate or low risk of bias. This is despite the fact that direct evidence for the comparisons is at high risk of bias. A highly quantitative approach would be to take a weighted average of ‘downgrading levels’, weighting by the contributions. For the CD comparison, we get (−1×0.041) + (−1×0.041) + (0×0.309) + (−1×0.35) + (−2×0.259) = −0.95 which also implies a downgrading of one level. Similar considerations for the other five network meta-analysis estimates lead us to downgrade for four of them for study limitations, but not to downgrade for the BD comparison because a large proportion of the information is from studies judged to be at low risk of bias.

### Indirectness

The standard GRADE guidance for indirectness considers (i) differences between the populations, treatments and outcomes in the studies to hand compared with the populations, treatments and outcomes targeted by the meta-analysis; and (ii) the use of indirect comparisons (5). The first set of issues is just as important when evaluating network meta-analysis estimates as when evaluating pairwise meta-analysis estimates. However, while we recognise the widespread concern about the validity of indirect comparisons, we argue against the idea of downgrading by default due to indirect evidence in the context of a network meta-analysis. Not only are indirect comparisons integral to the methodology of network meta-analysis, but under certain conditions indirect estimates can be just as reliable as direct comparisons (and in some probably uncommon situations even more reliable) [16]; [17].

A key point of our proposal for considering indirectness is that we recommend that the populations, treatments and outcomes be examined specifically for differences across different sources of direct evidence. The transitivity assumption underlying indirect comparisons, and hence network meta-analysis, requires that the distribution of effect modifiers is similar for all sources of direct evidence. We therefore propose that face validity of the transitivity assumption be assessed as part of the consideration of indirectness. In essence, the directness of each contributing source of direct evidence must be consistently close to the research question for the network meta-analysis to provide high quality evidence.

We advocate empirical comparison of the distribution of important effect modifiers across the comparisons present in the network. Unfortunately these effect modifiers can be unknown, unobserved or unreported in studies, so that transitivity might be difficult to judge. When similarity of these effect modifiers cannot be assured, researchers should downgrade due to concerns over intransitivity. Clinical understanding of the context, and familiarity with the trials to be synthesized, are necessary to infer about transitivity, particularly in the absence of data on effect modifiers. Other, conceptual approaches (e.g. by using directed acyclic graphs see [18]) can also be employed (for a review see [19]).

Each pairwise meta-analysis in the network can be evaluated following standard GRADE. Subsequently, the contributions of direct evidence to each pairwise network meta-analysis estimate can be taken into account, considering the most influential direct comparisons. To summarize the judgments from direct evidence, steps analogous to (b) to (d) described in *Study Limitations* can be followed.

### Application to antibiotics for discharging ears

The research question of this systematic review was to evaluate topical antibiotics (excluding steroids) for treating chronically discharging ears with underlying eardrum perforations. The authors of the review did not indicate that the studies were lacking relevance to their research question in terms of populations, treatments and outcomes. We are unable to undertake a detailed examination of the distribution of effect modifiers because most comparisons include few studies. As we lack convincing evidence for the plausibility of the transitivity assumption, we would recommend downgrading each pairwise comparison as well as the ranking of the treatments by one level.

### Inconsistency

In the usual GRADE approach, inconsistency refers to variability in the magnitude of effects across studies for a specific comparison that remains unexplained after accounting for important differences between subgroups. This variability is commonly known as heterogeneity. In the network meta-analysis context, the term inconsistency is frequently used specifically to refer to disagreement between direct and indirect evidence. For clarity, we will therefore use the term heterogeneity to describe disagreement between estimates within the same comparison and the term inconsistency for disagreement between estimates coming from different sources (e.g. direct and indirect evidence, or different routes of indirect evidence). We regard the two notions as very closely connected; inconsistency can be viewed as the extension of heterogeneity across studies evaluating different comparisons. Both are statistical consequences of between-study differences in populations, treatments, outcomes and biases, and inconsistency often appears as large heterogeneity in models that ‘force’ sources of evidence to be consistent. Thus we suggest joint consideration of both notions here. Some technical considerations are necessary before presenting our proposal to consider downgrading for inconsistency.

### Heterogeneity (between-study variance within a comparison)

In the majority of network meta-analysis applications, an assumption is made that every source of direct evidence has the same heterogeneity variance. That is, there is a single heterogeneity variance for the whole network, pertaining to every one of the direct comparisons. The assumption simplifies the analysis and allows for heterogeneity to be incorporated for direct comparisons with only one study. The assumption has implications for the estimation of effect sizes in the network meta-analysis, because any heterogeneity in one direct comparison gets propagated through the whole network. For example, a direct comparison that appears homogeneous when considered alone may have a non-zero heterogeneity variance imposed on it in the network meta-analysis if other evidence in the network displays heterogeneity. A full consideration of heterogeneity in network meta-analysis therefore should include both the magnitude of heterogeneity within each direct comparison *and* the magnitude of the single variance estimate if it is used.

The magnitude of a heterogeneity variance (often denoted τ^2^) can be difficult to interpret. For binary outcomes, we recommend referring to empirical distributions of heterogeneity values typically found in meta-analyses [20]. For example, low heterogeneity could be considered when the estimated τ^2^ is less than the 50% quantile of the empirical distribution, moderate heterogeneity for τ^2^ between 50% and 75% quantiles and large heterogeneity for τ^2^ larger than the 75% quantile. We provide these quantiles for meta-analyses based on odds ratios in Table S1.

### Inconsistency (differences between direct and indirect evidence in the network)

The estimates from network meta-analyses are valid only under the assumption of transitivity [3]; [19]. If this assumption does not hold for the network, or for parts of it, no joint analysis should be carried out. Lack of transitivity can manifest itself in the data as disagreement between direct and indirect estimates, usually called inconsistency, incoherence or incongruence in the literature. However, some degree of deviation might be present and considerations about the magnitude of inconsistency should be taken into account in downgrading the evidence. Note that inconsistency can only be detected for comparisons with mixed evidence, so only network meta-analysis estimates for such comparisons are subject to possible downgrading for inconsistency. In networks without closed loops of evidence (i.e. without mixed evidence), inconsistency cannot be assessed, but the underlying assumption of transitivity should always be considered as part of the considerations for indirectness.

There are several statistical approaches to evaluate network inconsistency; for a review see Dias et al [21]. A summary of approaches used to evaluate network inconsistency can be found in the Appendix S2. Approaches that focus on evaluating the involvement of a particular comparison in network inconsistency (such as the node-splitting approach or simple comparisons of direct and indirect estimates [22]; [23]) are more relevant to making judgements about (pairwise) network meta-analysis effect estimates than are global assessments of inconsistency in a whole network. For example, if a particular loop of evidence in the network is found to be inconsistent, downgrading applies primarily to effect estimates that involve this loop.

Our proposed procedure for considering whether to downgrade a particular network meta-analysis effect estimate under the GRADE component of inconsistency is as follows.

- Evaluate the extent of *heterogeneity* for each direct comparison including at least two studies, following the standard GRADE guidelines [24]. For dichotomous outcomes, we can also refer the estimated magnitude to the empirical distribution of heterogeneity variances specific to the type of outcome and types of treatments being compared (e.g. using Table S1).
- If a common heterogeneity variance is being assumed, evaluate the impact of this variance on each network meta-analysis estimate by comparing the heterogeneity variance from the direct evidence in step (a) with the heterogeneity variance from the network meta-analysis.
- Consider the magnitude of the heterogeneity estimate (or estimates) from the network meta-analysis for each effect size of interest. A particularly convenient way to do this is to look at predictive intervals for the effect in a new study of each comparison [14]; [25]; [26]. In the presence of important heterogeneity, the predictive interval is wide and includes effect sizes with importantly different implications for practice.
- Assess the involvement of each comparison in any *inconsistency* in the network, for example whether the direct estimate agrees with indirect estimates. Inconsistent loops and direct estimates that do not ‘fit’ with other pieces of evidence can be identified using statistical methods as described in the Appendix S2.
- Make a judgment about downgrading for heterogeneity and/or inconsistency based on steps (b), (c) and (d) above. This might start by judging heterogeneity as described in steps a) to c); if important heterogeneity is found, the evidence might be downgraded by one or two levels according to standard GRADE guidance. If heterogeneity is moderate or low, consideration of inconsistency would proceed as in step d). In case of moderate heterogeneity and inconsistency, the evidence might be downgraded by two levels. In case of low heterogeneity and inconsistency it might be downgraded by one level (or two levels in case inconsistency is substantial). If neither inconsistency nor heterogeneity are found, no downgrading is needed.

Statistical evaluation of inconsistency has very little power in the presence of substantial heterogeneity and hence step d) is conditional on observing low or moderate heterogeneity in the network.

### Application to antibiotics for discharging ears

Information about heterogeneity in the example network is reported in the last two columns of Table 1. Numbers of studies per direct comparison are generally small. The BC comparison, with seven studies, yields an estimate of 0.31 for the heterogeneity variance. The network meta-analysis assumed a common heterogeneity variance, which was estimated as 0.73 (0.36–0.98). Both the pairwise τ^2^ and the common network τ^2^ estimates are below the 50% quantile of the empirical distribution for a comparison of pharmacological vs placebo/control and a subjective outcome (persistent discharge in our case, see Table S1: 25% and 50% quantiles are 0.34 and 1.12 respectively) so we would characterize heterogeneity as moderate to low. However, the estimates of heterogeneity for BD and CD comparisons are imprecisely estimated so inference based on those values is particularly uncertain.

**Figure 4:**
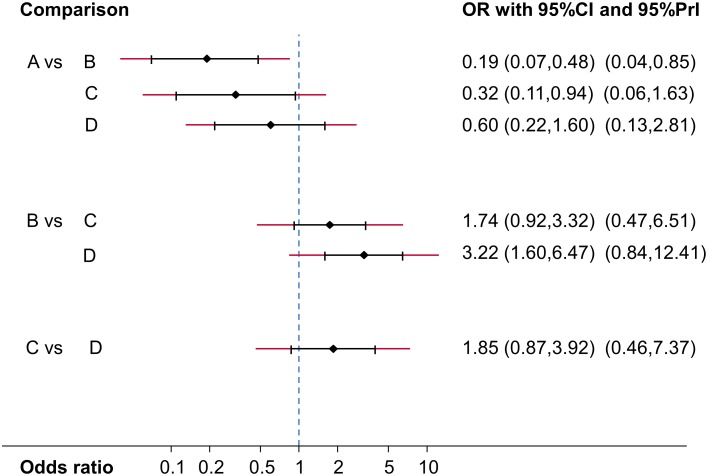
Network estimates of mean ORs, their 95% confidence intervals and 95% predictive intervals (red extensions). The names of the treatments are given in Figure 1.

In Figure 4 we present the mean effect sizes for the network estimates (OR) along with their confidence intervals and predictive intervals, all based on an assumption of a common heterogeneity variance. The predictive intervals for all comparisons except AB are compatible with either of the compared treatments being more effective. The predictive intervals for AC and BD potentially change the interpretation of the findings compared with the confidence intervals for the mean effects, since they extend across the line of OR = 1 when the confidence intervals for the means do not. We might therefore consider downgrading the network meta-analysis estimates for these comparisons.

The comparisons included in the network form two closed loops of evidence. For neither was there statistically significant evidence of inconsistency (discrepancy between direct and indirect evidence in the ABD loop is 1.56 on the log odds ratio scale, p = 0.10; and discrepancy in the BCD loop is 0.19, p = 0.81). However, power is low to detect important inconsistency and these results should not be interpreted as evidence of consistency; the point estimate for the ABD loop is very large suggesting that a true OR of 1 might be estimated to be 4.76.

In summary, we might want to downgrade two network estimates for AC and BD based only on our observations about heterogeneity and to consider downgrading evidence strongly influenced by studies involve in the ABD loop because of concerns over inconsistency in this loop (the point estimate might be considered large).

### Imprecision

In the standard GRADE guidance, imprecision is evaluated primarily by examination of 95% confidence intervals, and specifically whether these intervals exclude clinically relevant effect sizes. Rules of thumb are proposed for the consideration of appropriate sample sizes. In a network meta-analysis, we recommend focusing on the confidence intervals. Because of the complex contributions of different source of evidence to network meta-analysis estimate of effect size, convenient rules of thumb for considering sample sizes are not currently available. Otherwise, we suggest that the same criteria are applied to network meta-analysis estimates to decide whether downgrading by one or two levels (if any) is necessary.

### Application to antibiotics for discharging ears

To evaluate imprecision in the network estimates we consider the ORs and their confidence intervals presented in Figure 4. Applying the GRADE criteria, we suggest not to downgrade AB, AC and BD comparisons, but to downgrade AD, BC and CD by one level because their confidence intervals include values that favor either of the compared treatments.

### Publication bias

Even after a meticulous search for studies, publication bias can occur and usually it tends to lead to overestimation of an active treatment’s effect compared with placebo or other reference treatment. Several approaches have been proposed to generate assumptions about the presence of publication bias, including funnel plots, regression methods and selection models, but each has limitations and their appropriateness is often debated. Making judgements about the presence of publication bias in a network meta-analysis is usually difficult. We suggest that for each observed pairwise comparison, judgements about the presence of publication bias are made using standard GRADE. We recommend that the primary considerations are non-statistical (by considering how likely it is that studies may have been performed but not published) and we advocate the use of contour-enhanced funnel plots, which may help in identifying publication bias as a likely explanation of funnel plot asymmetry [27]. Then, judgements about the direct effects can be summarized to infer about the network estimates by taking into account the contributions of each direct piece of evidence.

### Application to antibiotics for discharging ears

The first consideration is the completeness of the search. The original review employed a comprehensive search strategy and authors report they have sought unpublished data. Pairwise comparisons include at most seven studies, so contour-enhanced funnel plots may not be very informative to infer about the possibility of publication bias. We do not recommend downgrading because of publication bias in this particular example.

## Grading the Quality of Evidence for Ranking of Treatments from a Network Meta-Analysis: Ranking GRADE

To determine our confidence in an overall treatment ranking from a network meta-analysis, we again follow the standard GRADE approach, making some modifications to reflect the same specific issues in network meta-analysis.

### Study limitations

The main consideration for study limitations in a network meta-analysis as a whole is again to ensure that the relative contributions of different sources of direct evidence (which may have different study limitations) are accounted for appropriately. Our proposed procedure is as follows.

- Evaluate each piece of direct evidence in the network and classify it as low, moderate or high risk of bias according to the usual GRADE guidelines [15]. It can be helpful to illustrate these assessments in a network plot, for instance by colouring lines corresponding to available direct estimates in different colours to reflect the judgements (e.g. green for low, yellow for moderate and red for high risk of bias). This can be done in STATA software (see [14]).
- Illustrate the risk of bias assessments according to the contributions of each source of direct evidence to the network meta-analysis as a whole, for example using a pie chart. For a formal statistical approach to this, we recommend using the contributions matrix as described in section *Study limitations.*
- Integrate the contributions and judgements of direct pieces of evidence into a single judgement about study limitations, and consider whether to downgrade the ranking evidence. A highly quantitative approach to this integration could also be employed.

### Application to topical antibiotics

**Figure 5:**
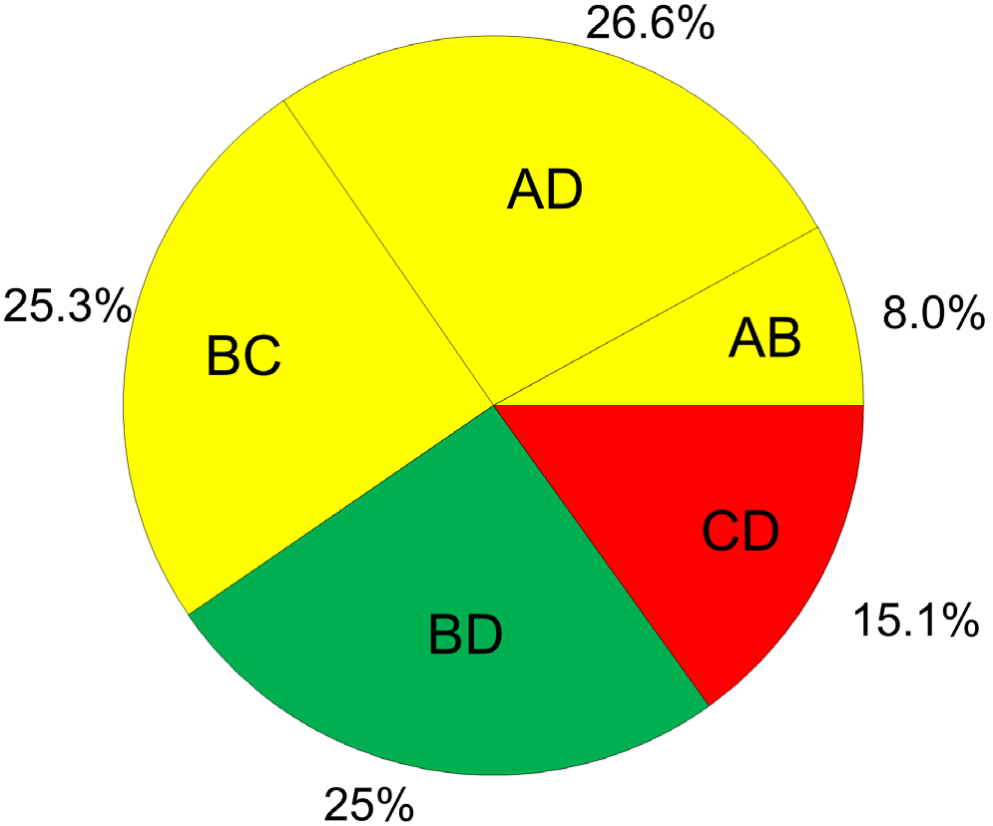
Study limitations weighted by contribution of direct estimates to the network of topical antibiotics. The colours represent the risk of bias (green: low, yellow: moderate, red: high). The names of the treatments are given in Figure 1.

Figure 5 presents the study limitation judgements accounting for the contribution of each piece of direct evidence to the whole network as presented in Figure 2. We can see that a judgement about study limitation for the ranking of the treatments is primarily derived (59.9%) from studies with moderate risk of bias and by 25% from studies with low risk of bias. For this reason we downgrade our confidence by one level for reasons of study limitations.

### Indirectness

We propose that judgments are made across all studies and all comparisons, considering potential differences between the populations, treatments and outcomes in the studies to hand compared with the populations, treatments and outcomes targeted by the network meta-analysis. This should include particular consideration of whether there are differences between studies making different comparisons, since such differences may invalidate transitivity assumptions made across the network. If some pieces of evidence only indirectly address the research question, then the quality of any treatment ranking is likely to be affected and we would consider downgrading for indirectness. Again, it would be possible to use the contributions matrix to describe the precise contribution of each direct estimate. Note however that it is possible for all of the evidence to be indirectly relevant to the research question but for it still to provide good evidence for a treatment ranking within a particular context, for example if all studies are in a particular sub-population (e.g. men) of a wider population of interest.

### Application to topical antibiotics

In the absence of evidence for an uneven distribution of effect modifiers, we decide that no downgrading is necessary for any of the direct comparisons, and consequently no downgrading of confidence for reasons of indirectness should take place for the overall ranking.

### Inconsistency

To assess inconsistency in the network as a whole, we again need to consider heterogeneity and network inconsistency. For the latter we suggest the implementation of statistical methods that evaluate the assumption of consistency in the entire network (e.g. comparisons of model fit, design-by-treatment global test, see Appendix S2). Our proposed procedure is as follows.

- Evaluate the extent of heterogeneity in the network. This is straightforward if a common heterogeneity variance is assumed. For dichotomous outcomes, we can refer to the empirical distribution of heterogeneity, as in section *Study limitations.*
- Evaluate inconsistency in the network as a whole, for instance using statistical methods that provide a single inference about the plausibility of assuming consistency throughout the network. The power of such global tests of inconsistency may be expected to be higher than local tests. However, power can still be low, and interpretation of the test result requires the usual caution. An alternative to a test is to estimate a global inconsistency parameter, such as the variance of the differences between direct and indirect evidence as described by Lu and Ades [28].
- Consider downgrading the confidence in the ranking by one or two levels depending on the presence and magnitude of heterogeneity and/or network inconsistency from steps (a) and (b). Network inconsistency is considerably more important than heterogeneity in assessing confidence in treatment rankings, because the ranks are based primarily on mean effects and so heterogeneity of effects around this mean may be less important.

### Application to topical antibiotics

To consider whether to downgrade confidence in the network as a whole due to inconsistency, we need to consider the network heterogeneity parameter and the presence of network inconsistency. A common heterogeneity variance was assumed in the analysis, with an estimated value that suggests the presence of moderate to low heterogeneity. The design-by-treatment interaction inconsistency model [29] produces a statistically significant test result for the presence of inconsistency in the network (P = 0.02). The ranking of the treatments could be downgraded by one level for reasons of both moderate heterogeneity and inconsistency.

### Imprecision

Imprecision in a ranking of treatments can be understood as uncertainty in the relative order of the treatments for the specific outcome. The ranking of treatments is often estimated by calculating ranking probabilities, with rankograms used to present the probability that each treatment is achieving a particular rank [8]. When rankograms illustrate similar distributions of ranks among the most and least effective options, this indicates an uncertain ranking. As extreme, hypothetical, examples of ranking imprecision, consider the results in Table 2 from four competing treatments. In both halves of the table the rank ordering is A-B-C-D. On the left hand side, each treatment has approximately 25% probability of being best, second, third and last in the ranking. On the right hand side, each treatment has almost 100% probability of being ranked at a specific place. The former leads to a highly imprecise ranking, and the latter to a highly precise ranking.

**Table 2:** Hypothetical (extreme) examples for ranking treatments with maximum (left) and minimal (right) imprecision.

	Network ranking with high imprecision				Network ranking with high precision			
	*A*	*B*	*C*	*D*	*A*	*B*	*C*	*D*
Best	28	24	24	24	97	1	1	1
Second	24	28	24	24	1	97	1	1
Third	24	24	28	24	1	1	97	1
Last	24	24	24	28	1	1	1	97

The entries in the table are the probabilities (as a percentage) of each treatment achieving each possible rank.

### Application to topical antibiotics

**Figure 6:**
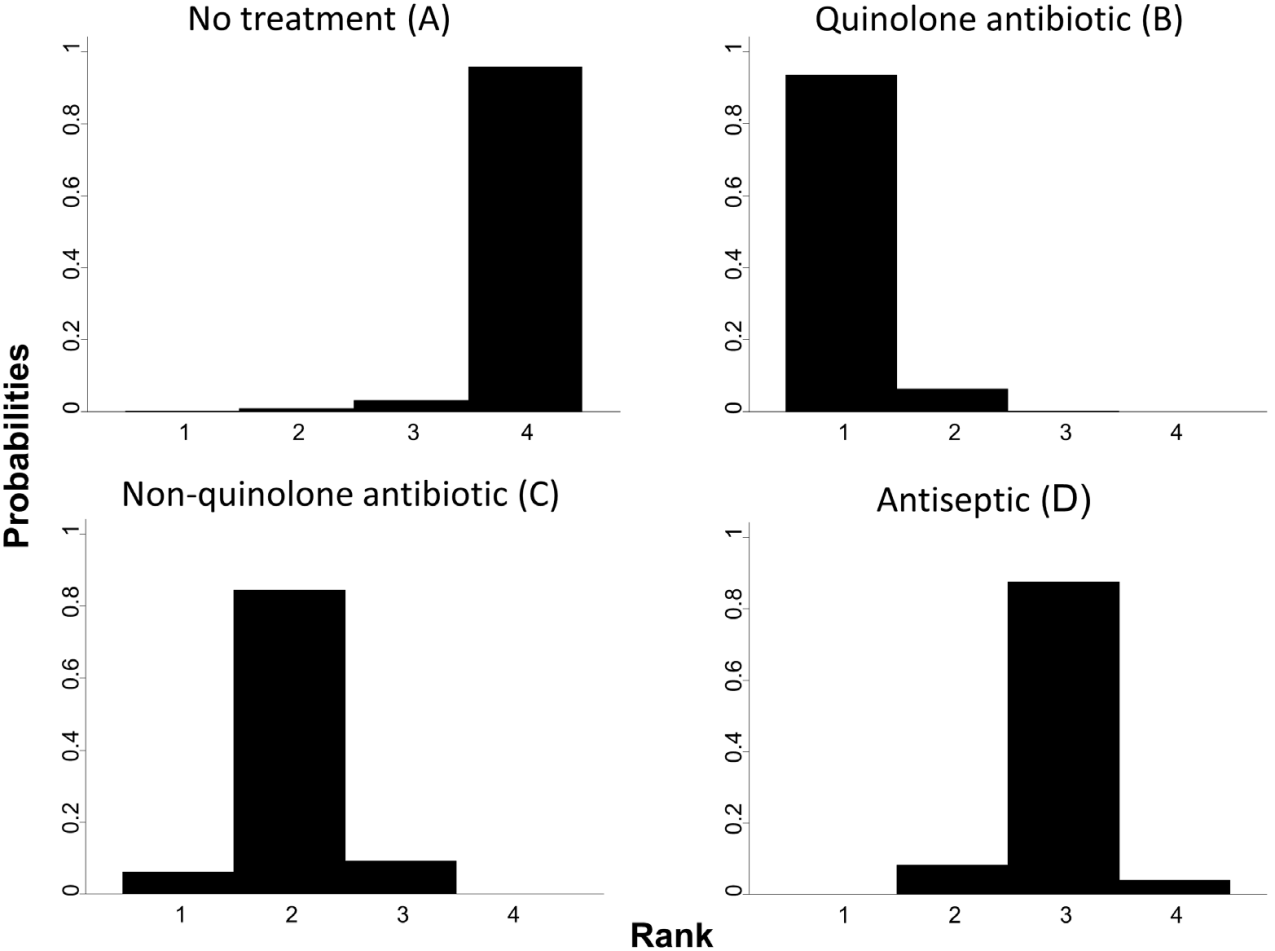
Rankograms for topical antibiotics without steroids for chronically discharging ears. On the horizontal axes are the possible ranks and on the vertical axis the probability that each treatment achieves each rank.

The rankograms for the example, illustrated in Figure 6, are reasonably precise and the probabilities for each treatment to rank low or high are well distinguished. Therefore we decide not to downgrade confidence in ranking for reasons of imprecision. Quinolone antibiotic (B) is clearly the best, non-quinolone antibiotic (C) is the second, antiseptic (D) the third and no treatment (A) the least effective.

### Publication bias

Judgments about the potential impact of publication bias in the ranking of the treatments require, as before, consideration of the comprehensiveness of the search for studies and the likelihood that studies may have been conducted and not published. A statistical approach to detecting bias is offered in certain situations by the *comparison-adjusted funnel plot* for a network of treatments [30]. In such a plot, the vertical axis represents the inverted standard error of the effect sizes as in a standard funnel plot. However, the horizontal axis represents an adjusted effect size, presenting the difference between each observed effect size and the mean effect size for the specific comparison being made. The use of such a plot is informative only when the comparisons can confidently be ordered in a meaningful way; for example, if all comparisons are of active treatment versus placebo, or all are of a new versus an old drug. Examination of any asymmetry in the plot can help to infer about the possible presence of an association between study size and study effect. Asymmetry does not provide evidence of publication bias, however, since associations between effect size and study size can be due to study limitations or genuine heterogeneity of effects.

### Application to topical antibiotics

**Figure 7:**
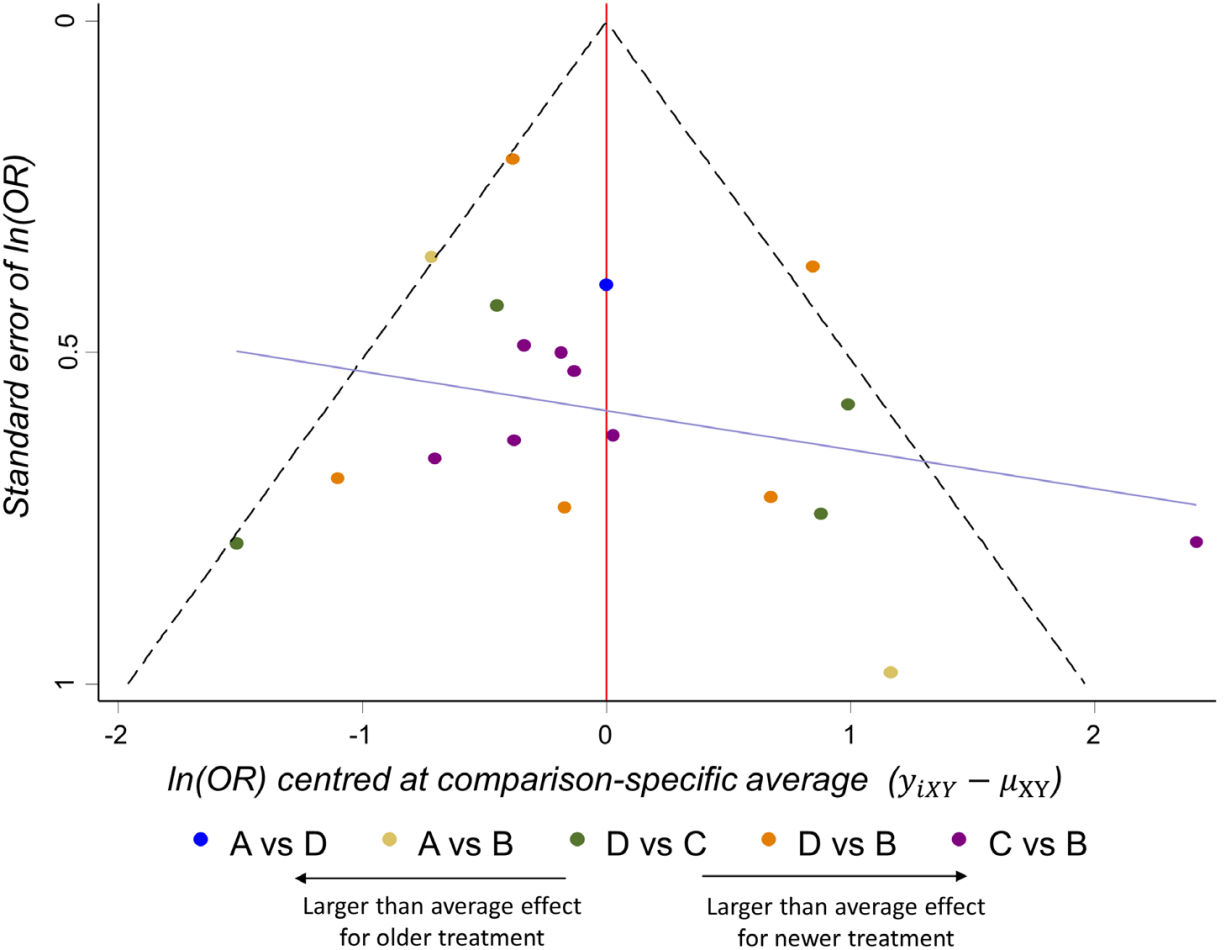
Comparison-adjusted funnel plot for the network of topical antibiotics without steroids for chronically discharging ears. Each observation is the difference between a study estimate and its direct meta-analysis mean effect. Studies on the right hand side ‘overestimate’ the effect of newer treatments.

The comparison-adjusted funnel plot in Figure 7 presents the centered ln (OR) for pairwise comparisons of a ‘reference’ treatment versus an active or newer or ‘experimental’ treatment [30]. We place the treatments in increasing order of their novelty or ‘activity’ (no treatment, antiseptic, non-quinolone antibiotic, quinolone antibiotic). We assume that publication bias, if present, is expected to exaggerate the effectiveness of the treatment that is ‘later’ in the order. For example, bias in a comparison of no treatment versus antiseptic meta-analysis might exaggerate the effectiveness of the antiseptic, but a comparison of antiseptic vs quinolone meta-analysis would exaggerate the effectiveness of quinolone. The comparison-adjusted funnel plot perhaps suggests a minor tendency of the smaller studies to emphasize the effectiveness of newer treatments. This observation should be taken into account and in combination with the confidence about the completeness of the search a judgment should be made for the entire network. We do not however suggest a downgrading of confidence for reasons of publication bias for this example.

## Discussion

We have proposed a strategy for considering the confidence of results from a network meta-analysis, building on ideas developed by the GRADE Working Group. At the heart of our proposal is the separation of an assessment for each pairwise estimate of treatment effect and for a ranking of treatments across the whole network. Both outputs are important and we summarize our suggested strategies in Table 3. In common with the standard GRADE process, we describe how to assess confidence for a specific outcome. In practice, clinical decisions involve trade-offs between benefits and harms, and the interpretation of relative effect sizes in the context of absolute event rates.

**Table 3:** Summary of domain assessment for evaluating the quality of evidence from a network meta-analysis: Procedures for a pairwise effect estimate and overall ranking.

Evaluate the confidence in a specific pairwise effect estimated in network meta-analysis			
GRADE domain	Domain assessment in NMA	Description of procedure	Instructions for downgrading
**Study Limitations**	Study limitations	Determine which directcomparisons contribute toestimation of the NMAtreatment effect^1^ and integraterisk of bias assessments fromthese into a single judgment.	Use standard GRADE considerations to inform judgment.
**Indirectness**	Joint considerationof indirectnessandintransitivity	Evaluate indirectness ofpopulations, interventions andoutcomes as in standardGRADE. Evaluate transitivityby comparing the distributionof known effect modifiersacross comparisons thatcontribute evidence toestimation of the NMAtreatment effect^1^.	If *a priori* assessment makes a transitivity assumption reasonable and suggests that effect modifiers are balanced, then do not downgrade. Otherwise downgrade (either if a transitivity assumption does not look reasonable or if there is insufficient evidence to judge).
**Inconsistency**	Joint considerationof statisticalheterogeneityand statisticalinconsistency	(a) Judge the extent ofheterogeneity, considering thecomparison-specificheterogeneity variance, theNMA estimate of variance, aprediction interval and/or otherrelevant metrics such as I^2^_._ (b)Evaluate the extent to which thecomparison under evaluationis involved in inconsistentloops of evidence.	(a) If important heterogeneity is found, downgrade. If heterogeneity is low do not downgrade. (b) Power to detect inconsistency may be low; Downgrade in absence of statistical evidence for inconsistency when direct and indirect estimates imply different clinical decisions.
**Imprecision**	Imprecision	Focus on width of theconfidence interval.	Assess uncertainty around the pairwise estimate. Downgrade if confidence interval crosses null value or includes values favoring either treatment).
**Publication bias**	Publicationbias	Non-statistical considerationof likelihood of non-publication ofevidence that would inform thepairwise comparison. Plot pairwiseestimates on contour-enhancedfunnel plot.	Use standard GRADE to inform judgment.
**Evaluate the confidence in treatment ranking estimated in network meta-analysis**			
**GRADE domain**	**Domain assessment in NMA**	**Description of procedure**	**Instructions for downgrading**
**Study Limitations**	Studylimitations	Integrate risk of bias assessmentsfrom each direct comparison toformulate a *single* overallconfidence rating for treatmentrankings.^1^	Use standard GRADEconsiderations to informjudgment.
**Indirectness**	Joint considerationof indirectnessand intransitivity	Evaluate indirectness of populations,interventions and outcomes as instandard GRADE. Evaluatetransitivity across network bycomparing the distribution of knowneffect modifiers acrosscomparisons.^1^	If *a priori* assessment of transitivity suggests effect modifiers are balanced across the network do not downgrade.Otherwise downgrade (either ifa transitivity assumption does not look reasonable or if thereis insufficient evidence to judge).
**Inconsistency**	Jointconsiderationof statisticalheterogeneityand statisticalinconsistency	(a) Judge the extent of heterogeneityconsidering primarily the NMAvariance estimate(s) used and othernetwork-wise metrics such as Q forheterogeneity in a network (b)Evaluate inconsistency in networkusing statistical methods (such as global tests of inconsistency, orglobal inconsistency parameter).	(a) If important heterogeneity is found, downgrade. If heterogeneity is low do not downgrade. (b) For overall treatment rankings, inconsistency should be given greater emphasis, since ranks are based on mean effects and the uncertainty they are estimated with. Downgrade in absence of statistical evidence for inconsistency when several direct and indirect estimates imply different clinical decisions.
**Imprecision**	Imprecision	Visually examine rankingprobabilities (e.g. rankograms) foroverlap to assess precision oftreatment rankings	If probabilities are similarly distributed across the ranks, downgrade for imprecision.
**Publication bias**	Publicationbias	Non-statistical consideration oflikelihood of non-publication foreach pairwise comparison. Ifappropriate, plot NMA estimateson a comparison adjusted funnelplot and assess asymmetry.	As asymmetry does not provideconcrete evidence of publication bias, downgrading should only be considered jointly with the non**-**statistical assessment.

1 When integrating assessments about direct comparisons into a judgement about an NMA treatment effect or the ranking, more weight should be given to assessments from direct comparisons that contribute more information. We recommend use of the contributions matrix to quantify how much information each direct comparison contributes to the estimation of the NMA treatment effect under evaluation or the ranking.

On application of our ideas to an example network of antibiotics for discharging ears, we found the suggestions to be workable, but subjective. Some of the subjectivity can be alleviated by taking a highly quantitative approach to considering the contributions of each piece of direct evidence, and weighting standard GRADE assessments for direct (pairwise) comparisons according to the influence they have on network meta-analysis estimates. There are advantages and disadvantages to the quantitative approach; it can be systematically applied, it is transparent and replicable, but it can be misinterpreted or over-interpreted. Furthermore, the quantitative measures of the contributions of each piece of direct evidence are only approximate when Bayesian methods are used for the network meta-analysis.

We have discussed each of the five GRADE domains and suggested possible strategies that can be used to form judgement for each domain separately. Decisions about downgrading by one or two levels for a specific GRADE component relate to the degree to which it compromises the summary estimate and the ranking. For instance, important inconsistency in the network can prompt investigators to downgrade the evidence by two levels. There is not a unanimously agreed definition of what consists ‘important’ inconsistency and, while tests and measures can be used to facilitate judgement, the potential to bias the summary estimate should be the primary consideration.

Table 4 summarizes the confidence we would have in the effect estimates from the network meta-analysis and in the ranking of the treatments. The domain-specific judgements should not be considered in isolation when an overall judgement is to be made about confidence in the evidence. The final rating of confidence is not necessarily obtained by aggregating the domain-specific judgements and may be different from the degree of downgrading suggested by the separate considerations for each domain. For instance, we had implicit rather than explicit concerns about intransitivity. Intransitivity could produce inconsistency, which however was not detected in the data possibly concealed by the large heterogeneity (which is also part of the inconsistency domain). Moreover, heterogeneity is responsible to a large degree for the low confidence in some comparisons due to imprecision. We considered these concepts jointly to derive the judgments presented in Table 4. We refer to guidance from the GRADE Working Group for more details about how the final rating of confidence can be derived [31].

**Table 4:** Summary of our confidence in effect estimates and ranking of treatments.

Comparison	Nature of the evidence	Confidence	Downgrading due to
AB: Quinolone antibiotic vs no treatment	Mixed	Low	Study limitations^1^; Indirectness^2^
AC: Non-quinolone antibiotic vs no treatment	Indirect	Low	Study limitations^1^; Inconsistency^3^
AD: Antiseptic vs no treatment	Mixed	Very low	Study limitations^1^; Imprecision^4^; Indirectness^2^
BC: Non-quinolone antibiotic vs quinolone antibiotic	Mixed	Very low	Study limitations^1^; Imprecision^4^; Indirectness^2^
BD: Antiseptic vs quinolone antibiotic	Mixed	Moderate	Inconsistency^3^
CD: Antiseptic vs non-quinolone antibiotic	Mixed	Very low	Study limitations^1^; Imprecision^4^; Indirectness^2^
*Ranking of treatments*		*Low*	*Study limitations* 5 *; Inconsistency* 6

1 Dominated by evidence at high or moderate risk of bias.

2 No convincing evidence for the plausibility of the transitivity assumption.

3 Predictive intervals for treatment effect include effects that would have different interpretations (there is additionally no convincing evidence for the plausibility of the transitivity assumption).

4 Confidence intervals include values favouring either treatment.

5 60% of the information is from studies at moderate risk of bias.

6 Moderate level of heterogeneity, and some evidence of inconsistency in the network.

None of the effect estimates was accompanied by high confidence, one had moderate confidence, three low confidence and one very low confidence. Notably, the one comparison for which there was no direct evidence was given low confidence, while one comparison that had been investigated in four studies was given very low confidence. Our confidence in the ranking of the four treatments is low, due to downgrading for study limitations and for inconsistency.

We have provided tables and figures that offer some possibilities for presenting GRADE assessments and the information that informs them. Figures 2 and 3 in particular illustrate the contributions of each direct comparison to the various network meta-analysis estimates of treatment effect. In practice we might expect to see GRADE assessments presented in an extended Summary of Findings table [7], but the optimum format for this is unclear. Presentation of results using a tabular format such as in Tables 1 and 4 becomes challenging with large networks that include many comparisons. Further work is needed to identify visual and numerical methods to present NMA results in a concise and informative way; a recent published review illustrates some useful alternatives [32]. Reporting guidelines for network meta-analysis are currently being developed.

Grading the evidence from a network meta-analysis assumes that the analysis is technically adequate. The assumption of transitivity is key to a network meta-analysis, and assessment of this assumption within the indirectness component of the GRADE framework is critical. Some degree of inconsistency might be present in the data and appropriate statistical methods should be employed to detect it. Investigators should refrain from network meta-analysis in the presence of important inconsistency. To account from small or moderate disagreement between the sources of evidence methods that encompass inconsistency should be employed to estimate effect sizes and ranking. However, particular care is needed when interpreting the results from such models.

## Supporting Information

Table S1 **Empirical distributions for the heterogeneity variance (τ^2^) of log odds ratios.**
(DOCX)

Appendix S1 **Estimation of the contribution of direct evidence to the network meta-analysis estimates.**
(DOCX)

Appendix S2 **Available approaches for the evaluation of network inconsistency.**
(DOCX)
