# Supplementary material for: Evaluating the Quality of Evidence from a Network Meta-Analysis
Source: PLoS One. 2014 Jul 3;9(7):e99682. doi: 10.1371/journal.pone.0099682 (PMC4084629; doi:10.1371/journal.pone.0099682)
Supplement: Table S1 — Empirical distributions for the heterogeneity variance (τ2) of log odds ratios. (DOCX) [file pone.0099682.s001.docx]

**Table S1. Empirical distributions for the heterogeneity variance (τ^2^) of log odds ratios**

| **Outcome type** | **Pharmacological *vs.* placebo** | **Pharmacological *vs.* pharmacological** | **Any non-pharmacological** |
| --- | --- | --- | --- |
|  | 50% quantile  75% quantile | 50% quantile  75% quantile | 25% quantile  50% quantile |
| **All-cause mortality** | 0.007  1.017 | 0.005  1.014 | 0.007  1.02 |
| **Semi-objective** | 0.014  1.05 | 0.011  1.04 | 0.016  1.058 |
| **Subjective** | 0.34  1.12 | 1.10  3.28 | 0.045  1.14 |
