# Supplementary material for: Evaluating the Quality of Evidence from a Network Meta-Analysis
Source: PLoS One. 2014 Jul 3;9(7):e99682. doi: 10.1371/journal.pone.0099682 (PMC4084629; doi:10.1371/journal.pone.0099682)
Supplement: Appendix S1 — Estimation of the contribution of direct evidence to the network meta-analysis estimates. (DOCX) [file pone.0099682.s002.docx]

**Appendix S1**

***Estimation of the contribution of direct evidence to the network meta-analysis estimates***

Here we describe an approach to determining the contribution of each piece of direct evidence to estimates from a network meta-analysis as suggested by [1;2]. We provide STATA code (netweight) to estimate the contributions of each direct piece of evidence at [www.mtm.uoi.gr](http://www.mtm.uoi.gr). R code is also available [1].

Suppose we have a network of $T$ treatments consisting of studies comparing pairs of treatments. We perform pairwise meta-analysis for all observed comparisons to obtain the direct, comparison-specific summary estimates $\hat{\mu}_{jk}^{D}$ for $j,k=1,\ldots,T, j\neq k$treatments. The direct treatment effects are estimated with variances$1/w_{jk}^{D}$.

A fixed-effect network meta-analysis under a consistency model is a linear model

$\hat{\mu}^{D}=X\mu$ (1)

where $\mu^{D}$ is the vector of the direct meta-analysis estimates$\mu_{jk}^{D}$, $\mu$is the set of $T-1$*basic parameters* that need to be estimated and $X$the design matrix which describes the linear combination between the basic parameters and the *functional parameters* that represent all other treatment comparisons. The basic parameters can be estimated using

$\hat{\mu}=(X'V^{-1}X)^{-1}X'V^{-1}\hat{\mu}^{D}$**,** (2)

where matrix $V$is the diagonal matrix of variance of the pairwise effect estimates. For example, in a triangle ABC where all three comparisons can be estimated using direct studies, this variance is

$V=\left(\begin{matrix} 1/w_{AB}^{D} & 0 & 0 \\ 0 & 1/w_{AC}^{D} & 0 \\ 0 & 0 & 1/w_{BC}^{D} \end{matrix}\right)$.

The basic parameters estimated in model (1) can be combined to produce the network estimates of $\mu^{N}$ which can be written as

$\hat{\mu}^{N}=X\hat{\mu}$.

Substituting $\hat{\mu}$ by equation (2), we obtain

$$\hat{\mu}^{N}=X(X'V^{-1}X)^{-1}X'V^{-1}\hat{\mu}^{D}$$

The matrix $H=X(X'V^{-1}X)^{-1}X'V^{-1}$is known as the *hat matrix*. The network estimates are therefore

$\hat{\mu}^{N}=H\hat{\mu}^{D}$ (3)

Equation (3) maps the direct estimates $\hat{\mu}^{D}$ to the network estimates $\hat{\mu}^{N}$ via the hat matrix. The hat matrix therefore describes the influence of each direct effect size on each network treatment effect estimate. It involves the direct weights $w_{jk}^{D}$ and the design matrix $X$, the latter of which reflects the network structure. The vector $\mu^{D}$ is the vector of the true treatment effects for the comparisons that are directly observed. It can be extended to have length equal the number $\left(\begin{matrix} T \\ 2 \end{matrix}\right)$ of all possible comparisons, even if some of them are not directly compared in trials. This can be done by simply imputing a small effect size with very large variance for those comparisons for which direct evidence is lacking. Consequently the $V$matrix will have dimension$\left(\begin{matrix} T \\ 2 \end{matrix}\right)\times\left(\begin{matrix} T \\ 2 \end{matrix}\right)$.

For the special case of a triangular network ABC where all comparisons have been studied in trials the ***H*** matrix is

$$H=\frac{1}{w_{AB}^{D}w_{BC}^{D}+w_{AB}^{D}w_{AC}^{D}+w_{AC}^{D}w_{BC}^{D}}\left(\begin{matrix} w_{AB}^{D}(w_{AC}^{D}+w_{BC}^{D}) & w_{AC}^{D}w_{BC}^{D} & -w_{AC}^{D}w_{BC}^{D} \\ w_{AB}^{D}w_{BC}^{D} & w_{AC}^{D}(w_{AB}^{D}+w_{BC}^{D}) & w_{AB}^{D}w_{BC}^{D} \\ w_{AB}^{D}w_{AC}^{D} & w_{AB}^{D}w_{AC}^{D} & w_{BC}^{D}(w_{AB}^{D}+w_{AC}^{D}) \end{matrix}\right)$$

The diagonal elements of the matrix show how much each direct estimate contributes to the network estimate for the same comparison; the off-diagonal entries show how much each direct comparison contributes indirectly to network estimates for a different comparison. The trace of the matrix equals the number of ‘basic’ parameters.

The rows of the ***H*** matrix can be re-expressed as percentages to show the relative contribution of each direct comparison to the network estimates (this is what is displayed in Figure 2). The sum of the columns can also be expressed as percentages to show the impact of each direct piece of evidence on the whole network.

Reference List

1. Krahn U, Binder H, Konig J (2013) A graphical tool for locating inconsistency in network meta-analyses. BMC Med Res Methodol 13(1):35.

2. Lu G, Welton NJ, Higgins JPT, White IR, Ades AE (2011) Linear inference for mixed treatment comparison meta-analysis: A two-stage approach. Res Synth Meth 2(1):43-60.
