# Supplementary material for: Evaluating the Quality of Evidence from a Network Meta-Analysis
Source: PLoS One. 2014 Jul 3;9(7):e99682. doi: 10.1371/journal.pone.0099682 (PMC4084629; doi:10.1371/journal.pone.0099682)
Supplement: Appendix S2 — Available approaches for the evaluation of network inconsistency. (DOCX) [file pone.0099682.s003.docx]

**Appendix S2**

***Available approaches for the evaluation of network inconsistency***

Inconsistency in a network of interventions refers to important differences between the direct and indirect estimates (or between indirect estimates through different intermediate treatments). Here we describe briefly the most popular methods that have been suggested for the evaluation of statistical inconsistency. These approaches are based either on statistical tests (e.g. *z*-test, *χ^2^*-test) or on alternatives to the standard network meta-analysis model to allow for discrepancies between different sources of evidence.

Perhaps the simplest approach is to estimate the (absolute) difference between a direct estimate $(D)$ and an indirect estimate $(I)$ of the same treatment effect, along with its uncertainty, for each (non-overlapping) closed loop in the network [1;2]. These differences are often called *inconsistency factors*. For example, in the triangular loop $ABC$ the inconsistency factor can be computed as $|\hat{D}_{BC}-\hat{I}_{BC}|$ with variance $\widehat{var}\left(D_{BC}\right)+\widehat{var}(I_{BC})$ , where$\hat{I}_{BC}=\hat{D}_{AC}-\hat{D}_{AC}$. One or more statistically significant inconsistency factors (judged by their 95% confidence interval not including the zero value) indicate that the consistency assumption might be invalid in the respective loops. In the presence of multi-arm studies, one of the comparisons is excluded so as not to overestimate consistency.

The ‘composite test for inconsistency’ suggested by Caldwell et al. [3] is similar to the inconsistency factor approach but allows for differences to be present also between the various indirect estimates derived from all possible independent loops in a network. The method estimates the (inverse variance) weighted average ($\tilde{d}$) of the direct ($\hat{D}_{1}$) and all the independent indirect estimates ($\hat{D}_{2}, \hat{D}_{3}, \ldots, \hat{D}_{N}$) and then the approximate $\chi^{2}$-statistic $T=\sum_{i=1}^{N} w_{i}\left(\hat{D}_{i}-\tilde{d}\right)^{2}$(where $w_{i}$ is the inverse variance of the estimate$\hat{D}_{i}$) is assumed to follow a $\chi_{N-1}^{2}$ distribution under the null hypothesis of consistency between all independent estimates. Inference about the presence of inconsistency is based on a $\chi^{2}$-test.

Another approach that takes into account the information from the entire network is the ‘node-splitting’ method suggested by Dias et al. [4] This method excludes each direct comparison from the network and each time estimates the indirect estimate for that comparison from the evidence in the rest of the network using standard network meta-analysis methods. The indirect estimates are then compared with the respective direct estimates in a similar manner to the inconsistency factor above, to infer about the presence of statistically significant inconsistency.

Measures of model fit and parsimony can be also employed to check the plausibility of the consistency assumption [5]. More specifically, both the consistency model and a simple inconsistency model (one in which the consistency equations have been omitted) are fitted. If the inconsistency model results in better fit and parsimony, this implies that the consistency assumption in the network might be violated.

Lu & Ades [6] suggested a network meta-analysis model that accounts for possible inconsistency by adding an extra term to the usual consistency equations to reflect the possible disagreement between the parameters representing the ‘true’ direct and indirect treatment effects ($\mu$) in a loop; namely for the $ABC$ loop the altered consistency equation would be:

$\mu_{CB}=\mu_{CA}-\mu_{BA}+w_{ABC}$

Usually, the inconsistency terms $w_{ijk}$ (where $i,j,k$are treatments of the network) are assumed exchangeable following a normal distribution with zero mean and common variance, hence$w_{ijk}\sim N(0,\sigma^{2})$.

An extension of this approach is the ‘design-by-treatment interaction’ model [7;8]. This model introduced a different possible form of inconsistency (known as design inconsistency) that can arise between the estimates coming from studies with different designs (e.g. two-arm vs. three-arm trials). In essence, the previous model is a special case of the design-by-treatment interaction model that omits the $w$terms reflecting design inconsistencies.

Finally, a test for the evaluation of inconsistency was recently proposed, which is based on the decomposition of the usual Q-statistic into two parts [9]; the sum of within-design Q-statistics (considered as a measure of heterogeneity) and the between-design Q-statistic (considered as a measure of inconsistency).

All presented methods have drawbacks, including the problem of multiple correlated tests in the inconsistency factor approach, or problems with dealing with multi-arm trials. A common issue for all suggested approaches is the low power they have to detect inconsistency and thus important inconsistency might be present even if we are not able to identify it [7].

Reference List

1. Song F, Harvey I, Lilford R (2008) Adjusted indirect comparison may be less biased than direct comparison for evaluating new pharmaceutical interventions. J Clin Epidemiol 61(5):455-463.

2. Veroniki AA, Vasiliadis HS, Higgins JPT, Salanti G (2012) Evaluation of inconsistency in networks of interventions. Int J Epidemiol 42(1):332-45.

3. Caldwell DM, Welton NJ, Ades AE (2010) Mixed treatment comparison analysis provides internally coherent treatment effect estimates based on overviews of reviews and can reveal inconsistency. J Clin Epidemiol 63(8):875-882.

4. Dias S, Welton NJ, Caldwell DM, Ades AE (2010) Checking consistency in mixed treatment comparison meta-analysis. Stat Med 29(7-8):932-944.

5. Dias S, Welton NJ, Sutton AJ, Caldwell DM, Lu G, et al (2013) Evidence synthesis for decision making 4: inconsistency in networks of evidence based on randomized controlled trials. Med Decis Making 33(5):641-656.

6. Lu G, Ades AE (2006) Assessing evidence inconsistency in mixed treatment comparisons. J Amer Stat Assoc 101.

7. Higgins JPT, Jackson D, Barrett JK, Lu G, Ades AE, et al (2012) Consistency and inconsistency in network meta-analysis: concepts and models for multi-arm studies. Res Syn Meth 3:98-110.

8. White IR, Barrett JK, Jackson D, Higgins JPT (2012) Consistency and inconsistency in network meta-analsyis: model estimation using multivariate meta-regression. Res Syn Meth 3:111-125.

9. Krahn U, Binder H, Konig J (2013) A graphical tool for locating inconsistency in network meta-analyses. BMC Med Res Methodol 13(1):35.
